# Supplementary material for: Potential of fecal microbiota for detection and postoperative surveillance of colorectal cancer
Source: BMC Microbiol. 2021 May 27;21:156. doi: 10.1186/s12866-021-02182-6 (PMC8157663; doi:10.1186/s12866-021-02182-6)
Supplement: Supplementary file 1 — Additional file 1 Table S1. OTUs stat of all groups. Table S2. Bacterial microbiota composition of each group at the genus level. Table S3. Feature list of LefSe analysis. Table S4. Survival state and basic information of 32 patients (paired samples). Figure S1. Venn of OTUs in all groups. Figure S2. Alpha diversity. Observed species, Chao and Ace reflected community richness; Shannon and Simpson reflected community diversity; Good’s coverage reflected the sequencing coverage. Figure S3. Microbiota composition of each group at the phyla level. The relative abundance less that 0.5% in all samples were combined as others. Figure S4. Microbiota composition of each group at the species level. The relative abundance less that 0.5% in all samples were combined as others. [file 12866_2021_2182_MOESM1_ESM.docx]

**Table S1 OTUs stat of all groups**

|  | Fa | Fb | NC |
| --- | --- | --- | --- |
| Sample n | 38 | 36 | 33 |
| Min OTU n | 56 | 27 | 92 |
| Max OTU n | 308 | 260 | 401 |
| Mean OTU (±SD） | 190.5±73.7 | 130.7±60.0 | 244.5±67.1 |

**Table S2 Bacterial microbiota composition of each group at the genus level**

| Taxon | Fa | Fb | NC |
| --- | --- | --- | --- |
| Bacteroides | 0.3233553 | 0.228916 | 0.3489311 |
| Other | 0.1845805 | 0.166014 | 0.1560167 |
| Escherichia | 0.1069364 | 0.185643 | 0.017645 |
| Faecalibacterium | 0.0549211 | 0.007359 | 0.0858073 |
| Prevotella | 0.0477655 | 0.023284 | 0.2148762 |
| Parabacteroides | 0.0383737 | 0.068063 | 0.0059306 |
| Fusobacterium | 0.0348777 | 0.032025 | 0.0003008 |
| Akkermansia | 0.0245388 | 0.037431 | 0.0014791 |
| Phascolarctobacterium | 0.0224098 | 0.006459 | 0.0073193 |
| Megamonas | 0.0222687 | 5.12E-05 | 0.0264367 |
| Ruminococcus | 0.0221821 | 0.027505 | 0.0301713 |
| Clostridium | 0.0205138 | 0.01398 | 0.0148241 |
| Veillonella | 0.0165846 | 0.004129 | 0.0007382 |
| Bifidobacterium | 0.0142193 | 0.005317 | 0.0094924 |
| Oscillospira | 0.0127759 | 0.00792 | 0.0063459 |
| Megasphaera | 0.0125637 | 0.001645 | 0.0005473 |
| Roseburia | 0.0100033 | 0.003841 | 0.0328248 |
| Pseudomonas | 0.0083752 | 0.011202 | 0.0000355 |
| Blautia | 0.0078228 | 0.001616 | 0.0071123 |
| Sutterella | 0.0067414 | 0.00353 | 0.0224923 |
| Dialister | 0.004987 | 0.001541 | 0.0102567 |
| Morganella | 0.0016505 | 0.046792 | 0.0000134 |
| Enterococcus | 0.001483 | 0.058167 | 0.0003229 |
| Acinetobacter | 6.827E-05 | 0.021133 | 7.838E-05 |
| Stenotrophomonas | 1.72E-06 | 0.036436 | 2.01E-06 |

**Table S3 Feature list of LefSe analysis**

| Biomarker | Logarithm value | groups | LDA score | P-value |
| --- | --- | --- | --- | --- |
| Bacteria.Fusobacteria.Fusobacteriia.Fusobacteriales.Fusobacteriaceae.Fusobacterium | 4.5642 | Fa | 4.2233 | 2.71E-07 |
| Bacteria.Firmicutes.Clostridia.Clostridiales.Veillonellaceae.Megasphaera | 4.1209 | Fa | 3.8634 | 0.006373 |
| Bacteria.Firmicutes.Clostridia.Clostridiales.Ruminococcaceae.Oscillospira | 4.2452 | Fa | 3.7680 | 0.009607 |
| Bacteria.Firmicutes.Clostridia.Clostridiales.Veillonellaceae.Acidaminococcus | 3.8544 | Fa | 3.5794 | 0.002237 |
| Bacteria.Firmicutes.Clostridia.Clostridiales.Lachnospiraceae.Blautia | 3.9529 | Fa | 3.5340 | 3.23E-07 |
| Bacteria.Firmicutes.Clostridia.Clostridiales.Tissierellaceae.Parvimonas | 3.1618 | Fa | 2.8919 | 2.61E-06 |
| Bacteria.Actinobacteria.Coriobacteriia.Coriobacteriales.Coriobacteriaceae.Collinsella | 3.4166 | Fa | 2.8680 | 0.007137 |
| Bacteria.Firmicutes.Erysipelotrichi.Erysipelotrichales.Erysipelotrichaceae.Bulleidia | 2.4685 | Fa | 2.3687 | 0.00015 |
| Bacteria.Firmicutes.Clostridia.Clostridiales.Lachnospiraceae.Lachnobacterium | 2.5433 | Fa | 2.3459 | 3.46E-08 |
| Bacteria.Proteobacteria.Gammaproteobacteria.Enterobacteriales.Enterobacteriaceae.Escherichia | 5.3149 | Fb | 4.9589 | 2.21E-06 |
| Bacteria.Bacteroidetes.Bacteroidia.Bacteroidales.Porphyromonadaceae.Parabacteroides | 4.8723 | Fb | 4.5449 | 0.002415 |
| Bacteria.Firmicutes.Bacilli.Lactobacillales.Enterococcaceae.Enterococcus | 4.8049 | Fb | 4.4965 | 2.69E-12 |
| Bacteria.Proteobacteria.Gammaproteobacteria.Enterobacteriales.Enterobacteriaceae.Morganella | 4.6980 | Fb | 4.4286 | 4.76E-05 |
| Bacteria.Proteobacteria.Gammaproteobacteria.Xanthomonadales.Xanthomonadaceae.Stenotrophomonas | 4.5283 | Fb | 4.1715 | 0.007819 |
| Bacteria.Firmicutes.Erysipelotrichi.Erysipelotrichales.Erysipelotrichaceae.Eubacterium | 3.8985 | Fb | 3.5270 | 1.04E-07 |
| Bacteria.Firmicutes.Clostridia.Clostridiales.Tissierellaceae.Finegoldia | 1.8628 | Fb | 3.3112 | 0.000734 |
| Bacteria.Actinobacteria.Actinobacteria.Actinomycetales.Actinomycetaceae.Actinomyces | 3.5457 | Fb | 3.2399 | 2.52E-07 |
| Bacteria.Proteobacteria.Gammaproteobacteria.Enterobacteriales.Enterobacteriaceae.Shigella | 1.4266 | Fb | 3.2021 | 0.00013 |
| Bacteria.Proteobacteria.Gammaproteobacteria.Enterobacteriales.Enterobacteriaceae.Yersinia | 3.5899 | Fb | 3.2015 | 0.005547 |
| Bacteria.Firmicutes.Clostridia.Clostridiales.Peptostreptococcaceae.Peptostreptococcus | 3.4937 | Fb | 3.1772 | 1.59E-08 |
| Bacteria.Firmicutes.Bacilli.Lactobacillales.Carnobacteriaceae.Granulicatella | 3.3126 | Fb | 3.0328 | 9.06E-07 |
| Bacteria.Actinobacteria.Coriobacteriia.Coriobacteriales.Coriobacteriaceae.Atopobium | 3.1918 | Fb | 2.8886 | 0.001358 |
| Bacteria.Actinobacteria.Actinobacteria.Actinomycetales.Corynebacteriaceae.Corynebacterium | 2.1291 | Fb | 2.7732 | 0.001681 |
| Bacteria.Actinobacteria.Coriobacteriia.Coriobacteriales.Coriobacteriaceae.Eggerthella | 3.0758 | Fb | 2.7113 | 5.81E-06 |
| Bacteria.Actinobacteria.Actinobacteria.Actinomycetales.Micrococcaceae.Rothia | 2.9697 | Fb | 2.6539 | 0.009373 |
| Bacteria.Firmicutes.Bacilli.Lactobacillales.Aerococcaceae.Abiotrophia | 2.8618 | Fb | 2.5707 | 0.003524 |
| Bacteria.Firmicutes.Clostridia.Clostridiales.Eubacteriaceae.Pseudoramibacter_Eubacterium | 2.9035 | Fb | 2.5650 | 1.52E-05 |
| Bacteria.Proteobacteria.Betaproteobacteria.Neisseriales.Neisseriaceae.Eikenella | 2.8374 | Fb | 2.5419 | 0.002865 |
| Bacteria.Firmicutes.Clostridia.Clostridiales.Mogibacteriaceae.Mogibacterium | 1.9769 | Fb | 2.3139 | 8.53E-05 |
| Bacteria.Bacteroidetes.Bacteroidia.Bacteroidales.Prevotellaceae.Prevotella | 5.3543 | NC | 4.9549 | 0.001556 |
| Bacteria.Firmicutes.Clostridia.Clostridiales.Ruminococcaceae.Faecalibacterium | 5.0110 | NC | 4.6587 | 3.91E-10 |
| Bacteria.Firmicutes.Clostridia.Clostridiales.Lachnospiraceae.Roseburia | 4.5880 | NC | 4.2357 | 7.99E-11 |
| Bacteria.Firmicutes.Clostridia.Clostridiales.Veillonellaceae.Megamonas | 4.4381 | NC | 4.2059 | 3.47E-06 |
| Bacteria.Proteobacteria.Betaproteobacteria.Burkholderiales.Alcaligenaceae.Sutterella | 4.4054 | NC | 4.0795 | 2.55E-09 |
| Bacteria.Firmicutes.Clostridia.Clostridiales.Lachnospiraceae.Coprococcus | 4.1063 | NC | 3.7730 | 8.55E-12 |
| Bacteria.Firmicutes.Clostridia.Clostridiales.Lachnospiraceae.Lachnospira | 4.0642 | NC | 3.7363 | 1.02E-13 |
| Bacteria.Firmicutes.Clostridia.Clostridiales.Veillonellaceae.Dialister | 4.0510 | NC | 3.6748 | 7.56E-05 |
| Bacteria.Firmicutes.Clostridia.Clostridiales.Lachnospiraceae.Defluviitalea | 1.3330 | NC | 3.5328 | 0.009918 |
| Bacteria.Firmicutes.Clostridia.Clostridiales.Ruminococcaceae.Gemmiger | 3.7376 | NC | 3.3212 | 6.46E-08 |
| Bacteria.Bacteroidetes.Bacteroidia.Bacteroidales.Paraprevotellaceae.Paraprevotella | 3.5312 | NC | 3.2350 | 1.83E-09 |
| Bacteria.Firmicutes.Bacilli.Bacillales.Thermoactinomycetaceae.Thermoactinomyces | 1.1820 | NC | 3.2155 | 0.000247 |
| Bacteria.Firmicutes.Erysipelotrichi.Erysipelotrichales.Erysipelotrichaceae.Allobaculum | 1.4070 | NC | 2.9040 | 0.00122 |
| Bacteria.Firmicutes.Clostridia.Clostridiales.Clostridiaceae.Sarcina | 1.3461 | NC | 2.9022 | 0.009918 |
| Bacteria.Firmicutes.Clostridia.Clostridiales.Lachnospiraceae.Anaerostipes | 2.9886 | NC | 2.6412 | 9.85E-08 |
| Bacteria.Actinobacteria.Coriobacteriia.Coriobacteriales.Coriobacteriaceae.Adlercreutzia | 2.3628 | NC | 2.5946 | 3.57E-12 |
| Bacteria.Proteobacteria.Betaproteobacteria.Burkholderiales.Comamonadaceae.Comamonas | 1.8375 | NC | 2.5262 | 5.37E-07 |
| Bacteria.Firmicutes.Clostridia.Clostridiales.Ruminococcaceae.Butyricicoccus | 2.5880 | NC | 2.5088 | 1.80E-08 |
| Bacteria.Firmicutes.Clostridia.Clostridiales.Clostridiaceae.02d06 | 2.1963 | NC | 2.4993 | 0.000325 |
| Bacteria.Firmicutes.Bacilli.Turicibacterales.Turicibacteraceae.Turicibacter | 2.5207 | NC | 2.4610 | 2.98E-07 |
| Bacteria.Firmicutes.Clostridia.Clostridiales.Veillonellaceae.Mitsuokella | 2.6093 | NC | 2.4438 | 6.51E-11 |
| Bacteria.Firmicutes.Clostridia.Clostridiales.Clostridiaceae.SMB53 | 2.5544 | NC | 2.3772 | 3.77E-07 |
| Bacteria.Lentisphaerae.Lentisphaeria.Victivallales.Victivallaceae.Victivallis | 2.1348 | NC | 2.2153 | 1.28E-06 |

**Table S4 Survival state and basic information of 32 patients (paired samples)**

| SampleID | Sex | Age | Time of operation | Survival state of now | Time of death or recidivation |
| --- | --- | --- | --- | --- | --- |
| A118 | female | 69 | 2017/6/10 | survival | - |
| A122 | female | 69 | 2017/6/15 | survival | - |
| A125 | female | 56 | 2017/6/22 | survival | - |
| A143 | female | 59 | 2017/5/22 | survival | - |
| A145 | female | 73 | 2017/5/19 | survival | - |
| A011 | female | 48 | 2016/11/2 | survival | - |
| A119 | male | 62 | 2017/6/14 | death | 20180823 |
| A124 | male | 63 | 2017/6/21 | survival | - |
| A127 | male | 62 | 2017/6/22 | survival | - |
| A136 | male | 52 | 2017/5/3 | survival | - |
| A138 | female | 59 | 2017/5/4 | survival | - |
| A139 | male | 52 | 2017/5/5 | survival | - |
| A147 | male | 72 | 2017/5/24 | recidivation | 20190110 |
| A153 | male | 87 | 2017/6/9 | survival | - |
| A155 | male | 71 | 2017/6/22 | survival | - |
| A158 | male | 75 | 2017/7/12 | survival | - |
| B101 | male | 55 | 2017/7/14 | survival | - |
| B102 | female | 44 | 2017/7/12 | survival | - |
| B103 | female | 85 | 2017/7/12 | death |  |
| B104 | female | 56 | 2017/7/21 | survival | - |
| B105 | female | 35 | 2017/7/17 | survival | - |
| B109 | male | 62 | 2017/7/27 | survival | - |
| B112 | male | 67 | 2017/8/1 | death |  |
| B123 | female | 57 | 2017/8/18 | survival | - |
| B126 | male | 63 | 2017/8/25 | survival | - |
| B106 | male | 71 | 2017/7/25 | death |  |
| B114 | male | 62 | 2017/8/9 | survival | - |
| B117 | male | 68 | 2017/8/11 | survival | - |
| B122 | male | 70 | 2017/8/16 | survival | - |
| B124 | male | 74 | 2017/8/22 | survival | - |
| B127 | male | 70 | 2017/8/24 | survival | - |
| B129 | male | 66 | 2017/8/31 | survival | - |


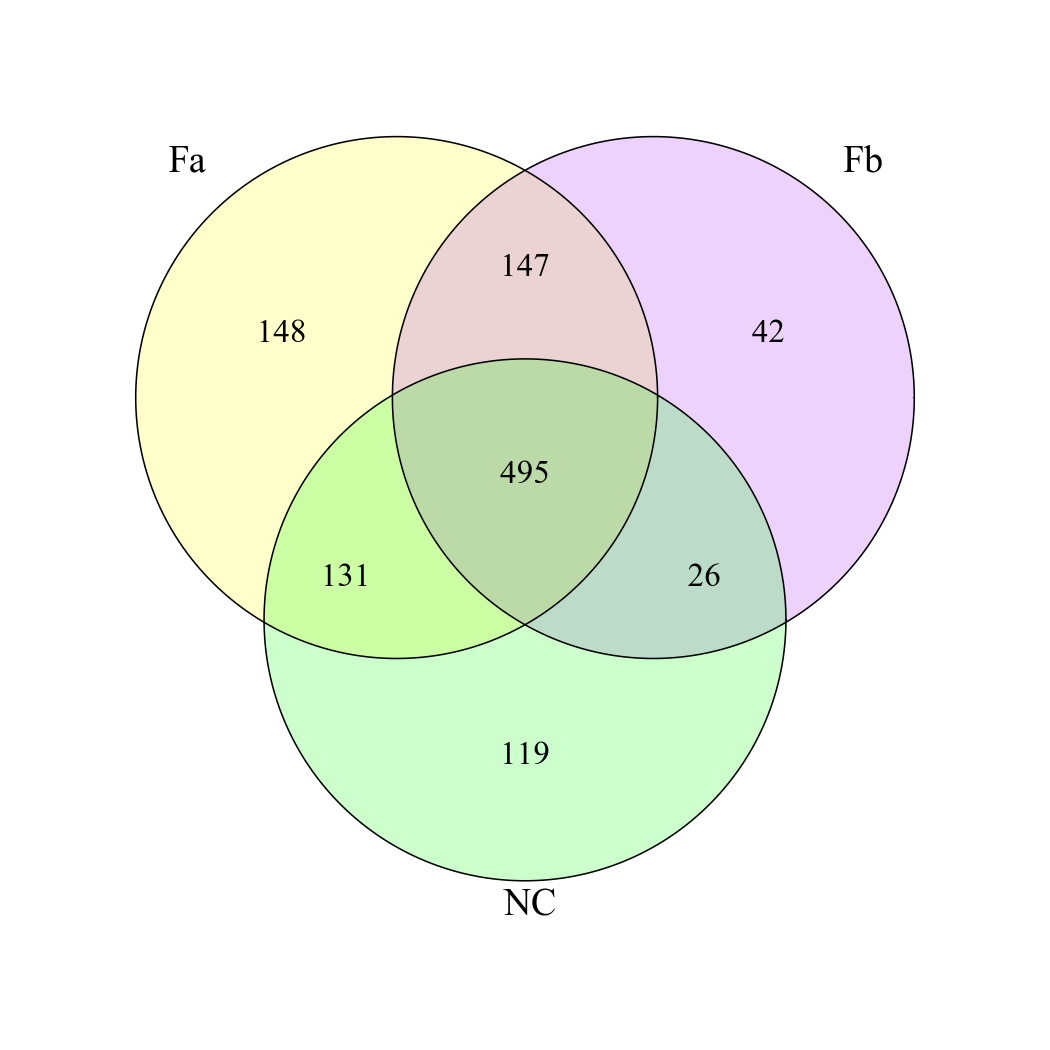


**Figure S1**. Venn of OTUs in all groups.


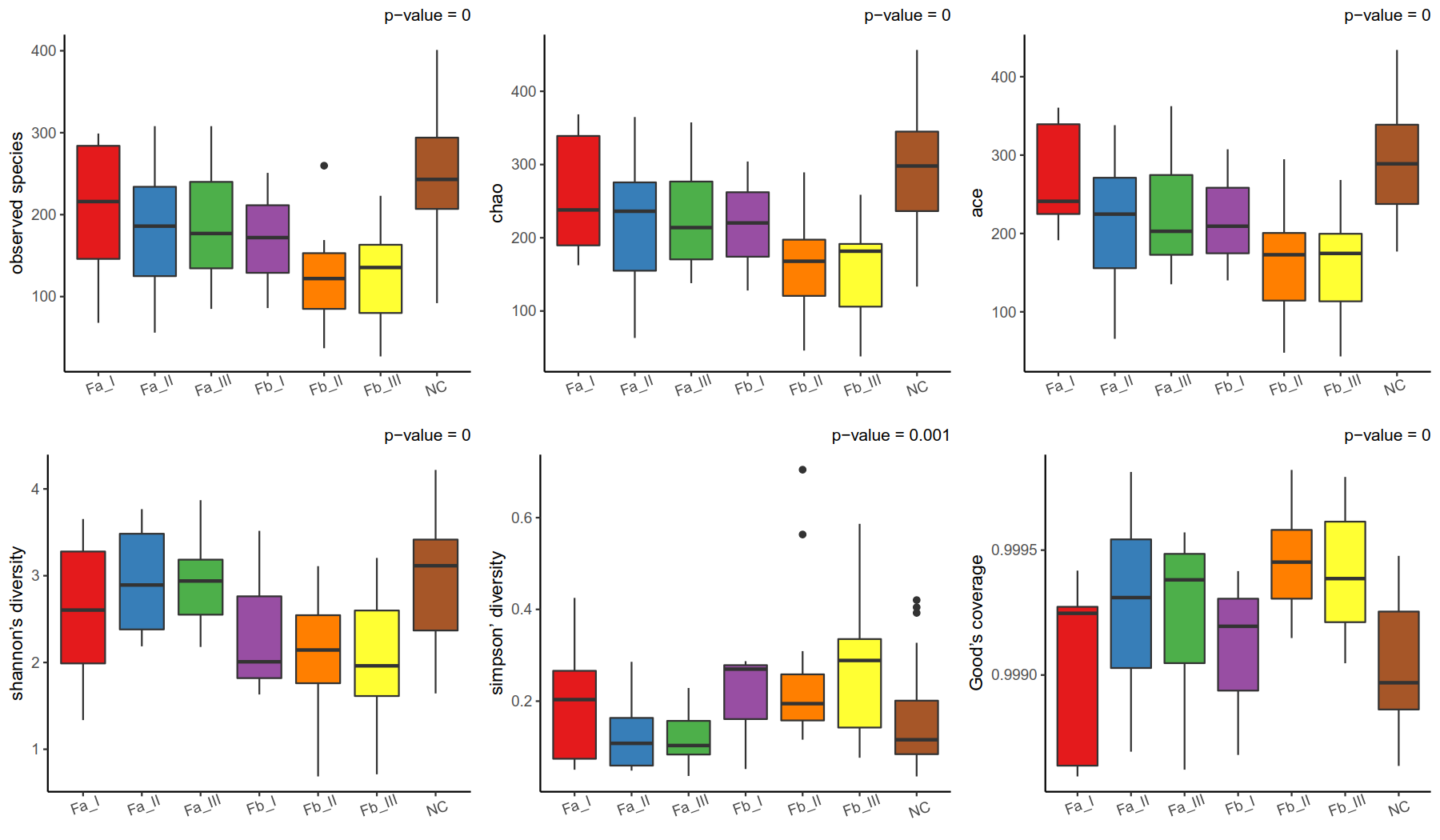


**Figure S2**. Alpha diversity. Observed species, Chao and Ace reflected community richness; Shannon and Simpson reflected community diversity; Good’s coverage reflected the sequencing coverage.


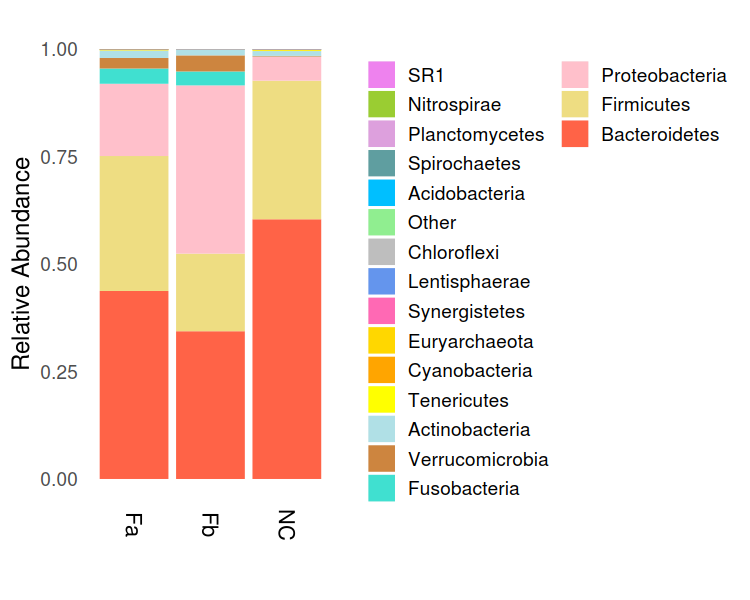


**Figure S3**. Microbiota composition of each group at the phyla level. The relative abundance less that 0.5% in all samples were combined as others.


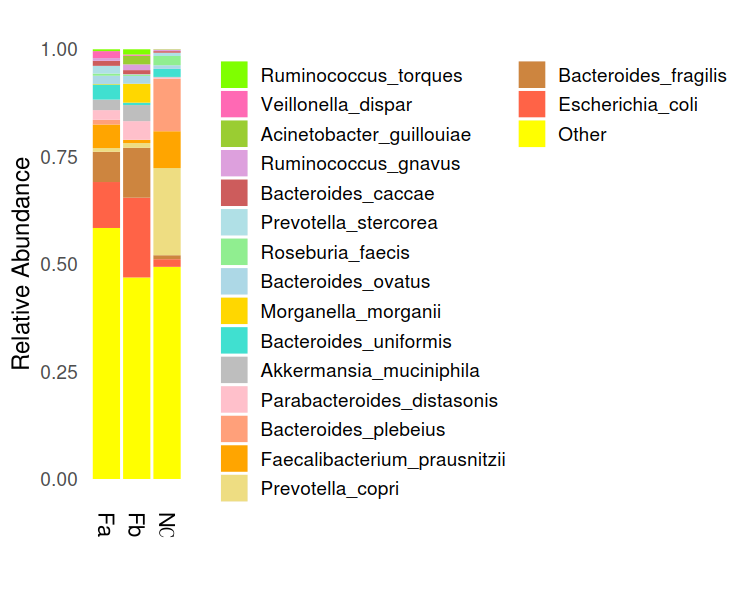


**Figure S4**. Microbiota composition of each group at the species level. The relative abundance less that 0.5% in all samples were combined as others.
